# Supplementary material for: Direct observation of autoubiquitination for an integral membrane ubiquitin ligase in ERAD
Source: Nat Commun. 2024 Feb 13;15:1340. doi: 10.1038/s41467-024-45541-3 (PMC10864399; doi:10.1038/s41467-024-45541-3)
Supplement: Supplementary file 1 — Supplementary Information [file 41467_2024_45541_MOESM1_ESM.pdf]

Direct observation of autoubiquitination for an integral membrane ubiquitin ligase in ERAD

Basila Moochickal Assainar[1], Kaushik Ragunathan[2]*, Ryan D. Baldridge[1,3]*

[1] Department of Biological Chemistry, University of Michigan Medical School, 1150 W Medical Center Drive, Ann Arbor, MI 48109, USA

[2] Department of Biology, Brandeis University, 415 South Street, Waltham, MA 02453, USA

[3] Cellular and Molecular Biology Program, University of Michigan Medical School, 1150 W Medical Center Drive, Ann Arbor, MI 48109, USA

*Correspondence: ryanbald@umich.edu and kaushikr@brandeis.edu

Supplementary Fig. 1. Validation of an improved Hrd1 labeling strategy.

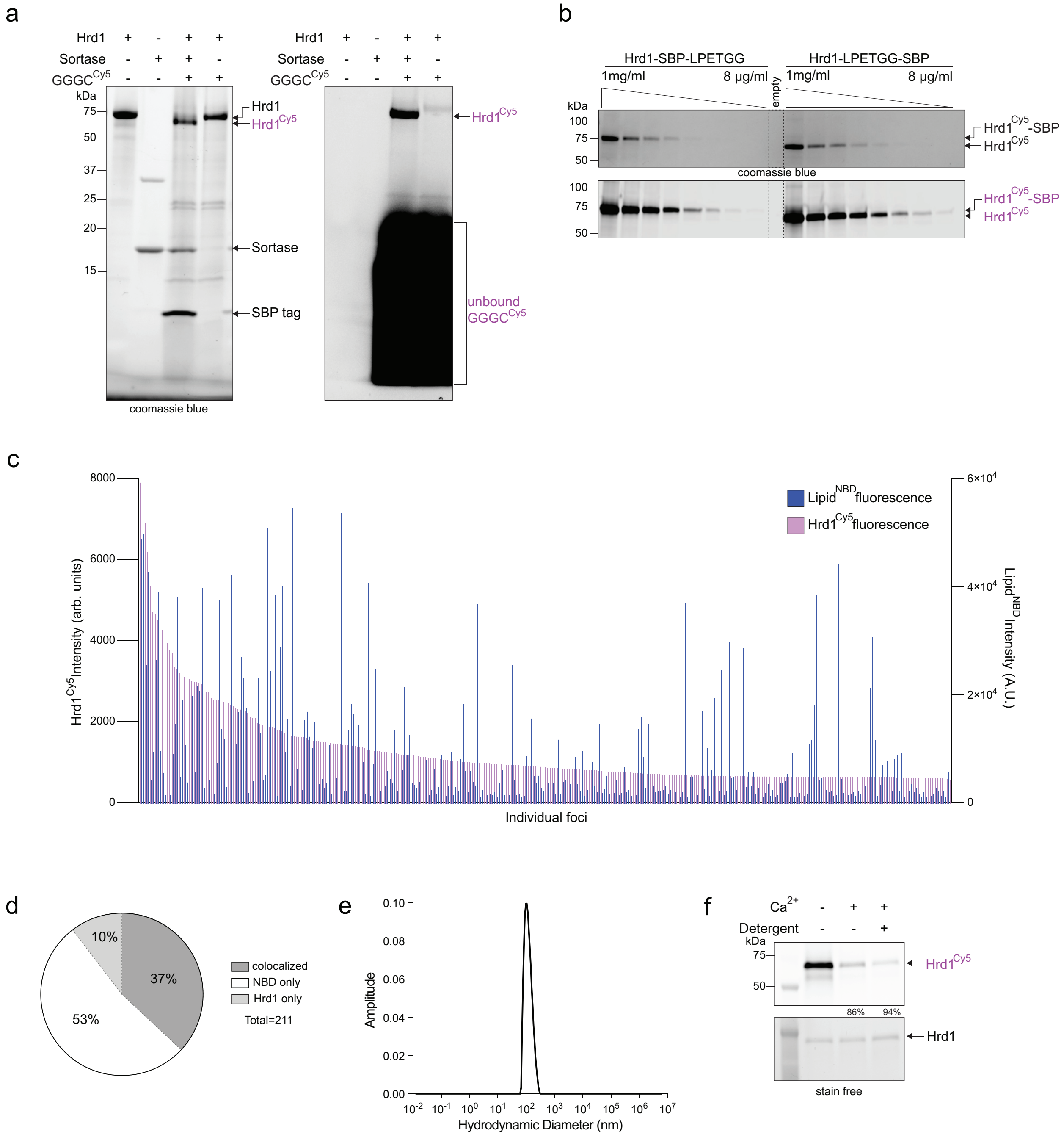

Supplementary Fig. 1 Validation of an improved Hrd1 labeling strategy. Related to Figure 1.

a) Uncropped SDS-PAGE image from Fig. 1a showing additional components of the labeling reaction including sortase A, the cleaved SBP tag, and uncoupled $GGGC^{Cy5}$ peptide.

b) Comparison of the Hrd1 Cy5 labeling efficiency for Hrd1-SBP-LPETGG (previous method) versus Hrd1-LPETGG-SBP (updated method).

c) Paired fluorescence intensities of $Hrd1^{Cy5}$ and the corresponding $lipid^{NBD}$. Proteoliposome foci were sorted based on $Hrd1^{Cy5}$ intensities.

d) Colocalization of $Hrd1^{Cy5}$ and $lipid^{NBD}$ in reconstituted proteoliposomes. The three categories correspond to liposomes containing both $Hrd1^{Cy5}$ and $lipid^{NBD}$, liposomes containing only $lipid^{NBD}$ and liposomes containing only $Hrd1^{Cy5}$.

e) Sizing of NBD-PC and $Hrd1^{Cy5}$ containing proteoliposomes reconstituted at 20:1 ratio using dynamic light scattering.

f) Orientation of $Hrd1^{Cy5}$ in proteoliposomes was tested through the accesibilty of the C-terminus by replacement of the C-terminal $GGGC^{Cy5}$ peptide with unlabelled GGGC peptide in the presence or absence of 10mM Ca^{2+} and/or 1% DMNG.
Each panel in this figure is representative of at least three independent experiments.

Supplementary Fig. 2. Characterization of the Poisson dilution based counting strategy.

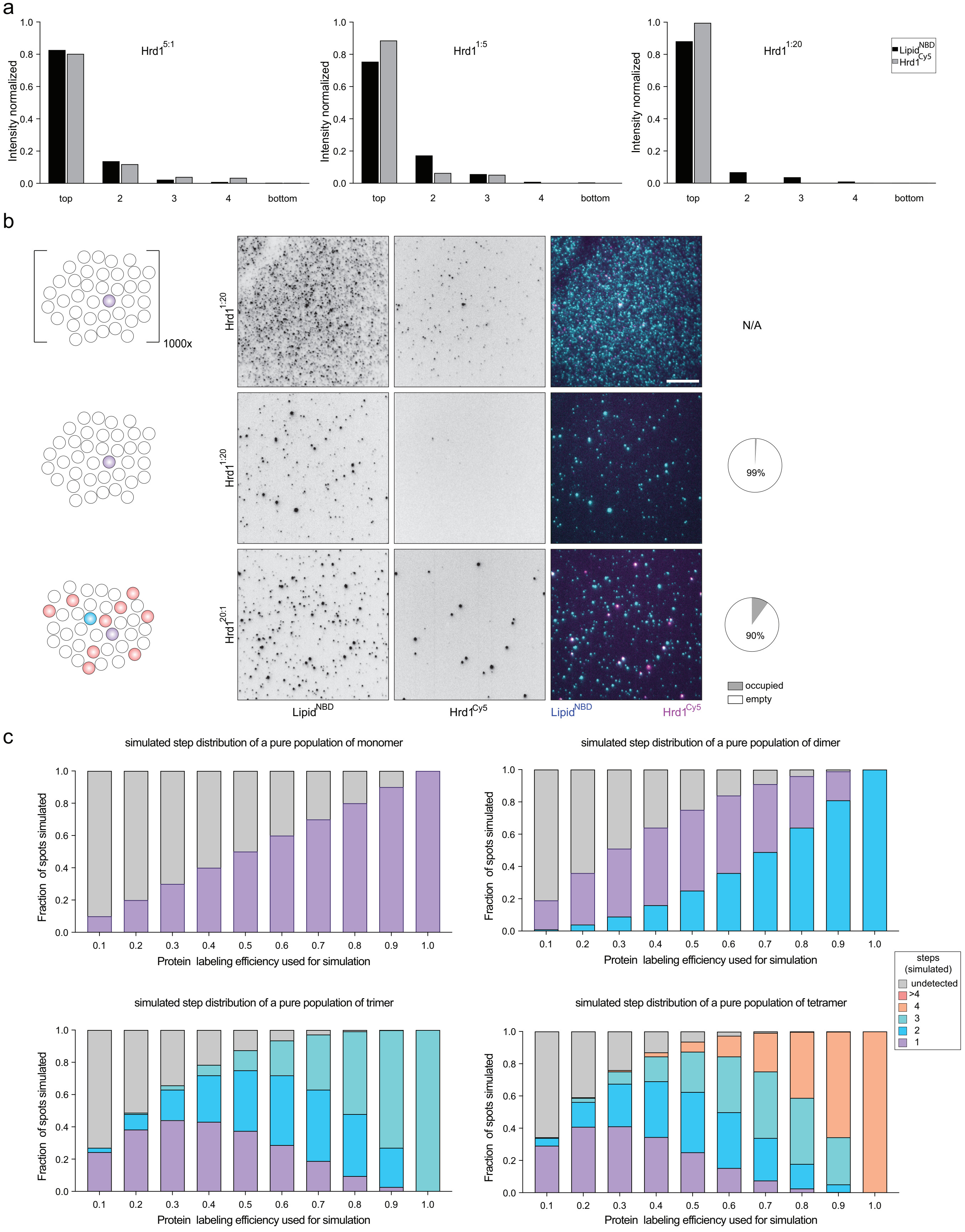

Supplementary Fig. 2. Characterization of the Poisson dilution based counting platform. Related to Figure 2.

a) Hrd1^{Cy5} proteoliposomes were reconstituted at three different protein:lipid ratios and floated using a glycerol density gradient. The gradient fractions were collected and analyzed using a fluorescence emission based plate reader to track the lipid and Hrd1 migration across the different glycerol layers. Fluorescence values for Hrd1^{Cy5} and lipid^{NBD} were normalized to their respective total intensity from all five fractions for each reconstitution condition. This figure is representative of three independent experiments.

b) Left: Schematic of reconstituted liposome experiment. Middle: NBD-PC (Lipid^{NBD}, left panels) and Hrd1^{Cy5} (center panels) proteoliposomes were visualized using TIRF excitation. Hrd1$^{1:20}$ proteoliposomes are shown in the top row and at a lower surface density in the middle row. Higher-occupancy Hrd1$^{20:1}$ proteoliposomes are shown in the bottom row. The right panels are overlay images to show colocalization of lipid^{NBD} (cyan) and Hrd1^{Cy5} (magenta). Each diffraction limited spot corresponds to an individual liposome. Right: The fraction of NBC-PC liposomes occupied by Hrd1, as counted from the middle images. The scale bar is 10 μm. This figure is representative of two independent experiments.

c) Simulated binomial distributions for monomeric, dimeric, trimeric or tetrameric complex plotted across varying protein labeling efficiencies displayed as simulated step distributions of various-sized complexes. The simulated step distribution in Fig. 2d represents the data corresponding to 90% labeling efficiency across these four separate graphs.

Supplementary Fig. 3. Ubiquitination reaction and correlation plots supporting the single-molecule ubiquitination experiments.

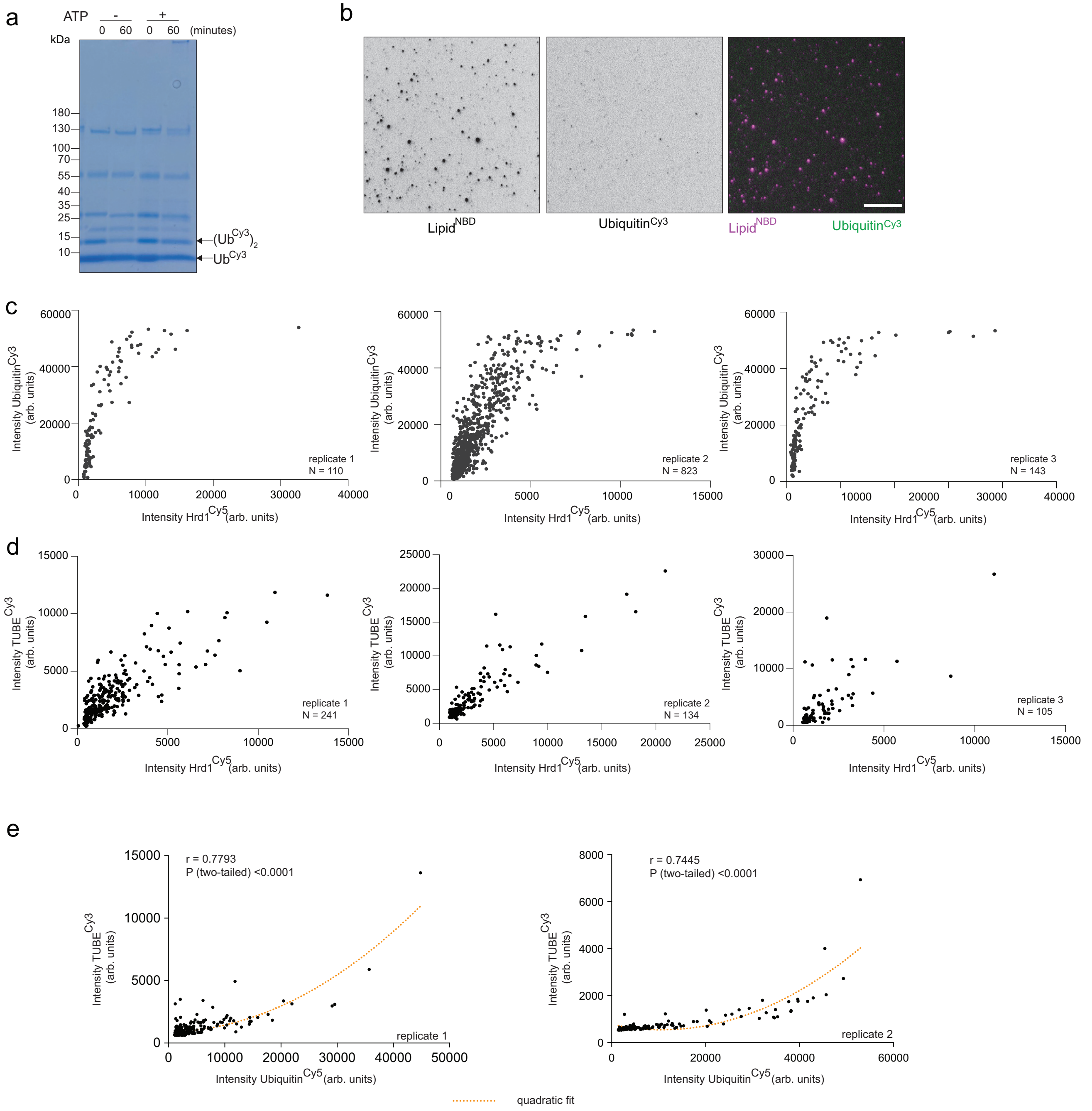

Supplementary Fig. 3. Ubiquitination reaction and correlation plots supporting the single-molecule ubiquitination experiments. Related to Figure 3.

a) Coomassie blue stained gel from Fig. 3a.

b) Empty liposomes containing NBD-PC (Lipid^{NBD}, left panels) were incubated with ubiquitin^{Cy3}, recombinant ubiquitin machinery, and ATP for 60 minutes. The reaction was immobilized on passivated coverslip surfaces and visualized under TIRF excitation. Lipid^{NBD} (left panels) and ubiquitin^{Cy3} (middle panels) images were overlaid with white showing colocalization (right panels). The scale bar is 10 µm.

c) Correlation plots of fluorescence intensities comparing Cy3 (fluorescent label on ubiquitin) and Cy5 (fluorescent label on Hrd1) for ubiquitinated Hrd1$^{20:1}$ proteoliposomes from three independent experiments.

d) Correlation plots of fluorescence intensities comparing Cy3 (fluorescent label on the tandem ubiquitin binding element, TUBE) and Cy5 (fluorescent label on Hrd1) for ubiquitinated Hrd1$^{20:1}$ proteoliposomes from three independent experiments.

e) Correlation plot of fluorescence intensities of Cy5 (fluorescent label on ubiquitin) and Cy3 (fluorescent label on TUBE) for ubiquitinated Hrd1$^{20:1}$ proteoliposomes from two independent experiments. Left panel: N= 256 foci within an independent reconstitution experiment. Right panel: N= 131 foci within an independent reconstitution experiment. Pearson correlation coefficient was calculated using GraphPad Prism.

Imaging panels in A and B are representative of at least three independent experiments.

Supplementary Fig. 4. Ubiquitination of individual Hrd1 and Hrd1(C399S) proteoliposomes.

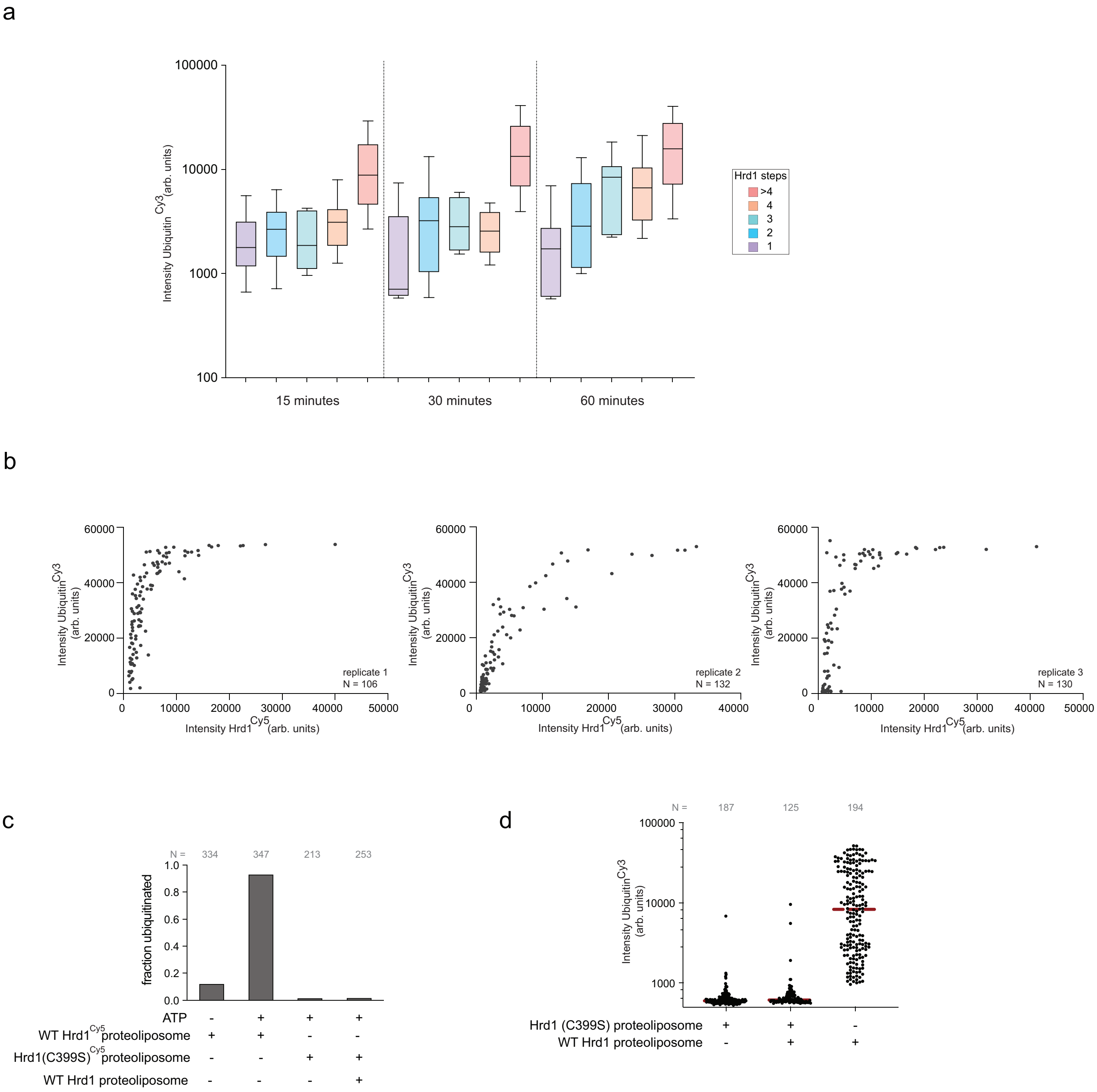

Supplementary Fig. 4. Ubiquitination of individual Hrd1 and Hrd1(C399S) proteoliposomes. Related to figure 4.

a) Ubiquitin^{Cy3} intensity at individual Hrd1^{Cy5} foci were separated by Hrd1 oligomer size in the presence of ubiquitination machinery and ATP based on the time of incubation with ATP. Boxes represent the median, 25th, and 75th percentiles, and the whiskers represent the 10th to 90th percentiles. Representative of three independent experiments.

b) Correlation plot of fluorescence intensities of Cy3 (fluorescent label on ubiquitin) and Cy5 (fluorescent label on Hrd1) for ubiquitinated Hrd1$^{20:1}$ proteoliposomes diluted with 20 fold excess of empty liposomes from three independent experiments.

c) Hrd1(C399S)^{Cy5} proteoliposomes (with biotinylated lipid) were incubated with ubiquitination machinery containing ubiquitin^{Cy3} and ATP +/- unlabeled wild-type Hrd1 proteoliposomes (with no biotinylated lipid). The reactions were immobilized on the slide surface and imaged using TIRF microscopy. The colocalization of Hrd1(C399S)^{Cy5} with ubiquitin^{Cy3} is displayed as fraction colocalized. Summary data combines two independent experiments for experiments with C399S, while three independent experiments for WT.

d) Quantification of ubiquitin^{Cy3} intensities for individual Hrd1(C399S)^{Cy5} proteoliposome foci either alone, or mixed with unlabeled wild-type Hrd1 proteoliposomes, that were incubated with ubiquitination machinery and ATP (quantified from the experiments in (c)). In the sample with unlabeled wild-type Hrd1 proteoliposomes only, the foci were selected based on Cy3 fluorescence intensity as from wild-type Hrd1 proteoliposomes non-specifically adhered to the slide surface. Representative of two independent experiments.

Supplementary Table 1. Plasmids used in this study

Plasmid name	Backbone, features	Reference	Figures
pBMA003	K27_His_{14}_SUMO_Cys_Ubiquitin (for maleimide labeling)	This study	Fig. 3,4
pRSET_6xTR_ TUBE	N-terminal His_6 -T7 tag (N terminal on insert), 6 tandem repeats of trypsin-resistant UBQLN1 UBA domain, all Arg residues in the UBA domain are substituted to Ala	46 Addgene #110313	Fig. 3, Supplementary Fig. 3
pBMA15	pRS426_GAL1prom_Hrd1_LPETGG_3C_SBP _Cyc1term	This study	Fig. 1,2,3,4, Supplementary Fig. 1,2,3,4
pBMA24	pRS426_GAL1prom_Hrd1(C399S)_LPETGG_ 3C_SBP_Cyc1term	This study	Supplementary Fig. 4
pET29_Sortase A_PentaMutant	pET29_sortase_A_pentamutant_His_6	26 Addgene #75144	Fig. 1, Supplementary Fig. 1
pAS153	pET28b_His_6_Ubc7	25	Fig. 3,4, Supplementary Fig. 3,4
pAS159	pET28b_His_6_Cue1(24-203) (truncated soluble Cue1 (24-203))	25	Fig. 3,4, Supplementary Fig. 3,4
pAS185	pRS426_GAL1prom_His_{14}_TEV_Uba1_Cyc1t erm	25	Fig. 3,4, Supplementary Fig. 3,4
